# Stratification of responses to tDCS intervention in a healthy pediatric population based on resting-state EEG profiles

**DOI:** 10.1038/s41598-023-34724-5

**Published:** 2023-05-25

**Authors:** Paulina Clara Dagnino, Claire Braboszcz, Eleni Kroupi, Maike Splittgerber, Hannah Brauer, Astrid Dempfle, Carolin Breitling-Ziegler, Alexander Prehn-Kristensen, Kerstin Krauel, Michael Siniatchkin, Vera Moliadze, Aureli Soria-Frisch

**Affiliations:** 1 Neuroscience BU, Starlab Barcelona SL, Av Tibidabo 47 bis, Barcelona, Spain; 2 Institute of Medical Psychology and Medical Sociology, University Medical Center Schleswig-Holstein, Kiel University, Kiel, Germany; 3 Department of Child and Adolescent Psychiatry, Center for Integrative Psychiatry Kiel, University Medical Center Schleswig-Holstein, Kiel, Germany; 4 Institute of Medical Informatics and Statistics, University Hospital Schleswig Holstein, Kiel University, Kiel, Germany; 5 Department of Child and Adolescent Psychiatry and Psychotherapy, University of Magdeburg, Magdeburg, Germany; 6 Clinic for Child and Adolescent Psychiatry and Psychotherapy, Protestant Hospital Bethel, University of Bielefeld, Campus Bielefeld Bethel, Bielefeld, Germany

**Keywords:** Predictive markers, Biomedical engineering, Neurophysiology

## Abstract

Transcranial Direct Current Stimulation (tDCS) is a non-invasive neuromodulation technique with a wide variety of clinical and research applications. As increasingly acknowledged, its effectiveness is subject dependent, which may lead to time consuming and cost ineffective treatment development phases. We propose the combination of electroencephalography (EEG) and unsupervised learning for the stratification and prediction of individual responses to tDCS. A randomized, sham-controlled, double-blind crossover study design was conducted within a clinical trial for the development of pediatric treatments based on tDCS. The tDCS stimulation (sham and active) was applied either in the left dorsolateral prefrontal cortex or in the right inferior frontal gyrus. Following the stimulation session, participants performed 3 cognitive tasks to assess the response to the intervention: the Flanker Task, N-Back Task and Continuous Performance Test (CPT). We used data from 56 healthy children and adolescents to implement an unsupervised clustering approach that stratify participants based on their resting-state EEG spectral features before the tDCS intervention. We then applied a correlational analysis to characterize the clusters of EEG profiles in terms of participant’s difference in the behavioral outcome (accuracy and response time) of the cognitive tasks when performed after a tDCS-sham or a tDCS-active session. Better behavioral performance following the active tDCS session compared to the sham tDCS session is considered a positive intervention response, whilst the reverse is considered a negative one. Optimal results in terms of validity measures was obtained for 4 clusters. These results show that specific EEG-based digital phenotypes can be associated to particular responses. While one cluster presents neurotypical EEG activity, the remaining clusters present non-typical EEG characteristics, which seem to be associated with a positive response. Findings suggest that unsupervised machine learning can be successfully used to stratify and eventually predict responses of individuals to a tDCS treatment.

## Introduction

In recent years, Non Invasive Brain Stimulation (NIBS) techniques and, in particular, transcranial Direct Current Stimulation (tDCS)^1,2^, have been successfully used to study brain function, treat mental disorders and enhance cognitive functions in adults. TDCS therapy can be applied as an effective treatment option^3,4^, which is particularly interesting in the absence of alternative interventions. Its application in paediatric populations however has been limited due to initial concerns^5^. In children and adolescents the stimulation parameters cannot be directly translated from studies made in adult populations since the electric fields generated in the brain during stimulation are affected by anatomical differences such as brain volume, skull thickness, white matter - gray matter ratio and cerebrospinal fluid (CSF) volume^6^.

Attention-Deficit Hyperactivity Disorder (ADHD) is one of the most prevalent neurodevelopmental conditions, which affects 5.9% of youth and 2.5% of adults (in America, Europe, Australia, and Asia)^7^. Electroencephalography (EEG) has been used to characterize the clinical population with an ADHD diagnosis. The use of EEG-based markers overcomes some limitations of classical subjective assessments based on interviews and physical examinations. Existing EEG biomarkers include an increased theta band power and decreased beta band power in the frontocentral brain region^8,9^, which is generally known as slowing, phase-amplitude coupling (beta-gamma) deficits in the frontal-left hemisphere^10^, and modified peak alpha frequency^11^ among others.

Given the concerns that drug-based treatments of ADHD have raised in the past, there is an ongoing search for treatments with reduced or no side effects^12^. Current drug-based treatments and behavioral therapy in ADHD are also challenged by the existence of different phenotypes and related symptoms of the pathology, as well as the complexity associated with the developing brain^13^. TDCS interventions for ADHD constitute an interesting alternative to drug treatments^14^. However, an important factor to take into account in the development of efficient tDCS interventions is the inter-subject variability of the effects of tDCS^15,16^. Here, machine learning techniques have been proposed to identify subgroups with differential tDCS responses based on the clustering of behavioral responses during tDCS interventions^15^. Moreover, other works have implemented unsupervised methods to characterize responses to different treatments based on the clustering of genetic^17^, clinical^18^ and functional Magnetic Resonance Imaging (fMRI)^19^ data. These works report the presence of patient subgroups within an a priori uniform group of patients. We propose to perform patient stratification based on EEG data. Previous work using EEG data attains the prediction of response to treatment through supervised Machine Learning (ML) approaches^20,21^. Variability in response to TMS and tDCS interventions in patients suffering from depression or anxiety can be also explained by individual differences in baseline EEG activity^22,23^. EEG is an attractive modality for clinical application due to its lower cost and faster application time than other neuroimaging modalities. In the present study, we have developed an unsupervised clustering method applied to EEG data to extract so-called digital phenotypes^24^, making use of EEG for the first time to stratify tDCS response in a healthy pediatric population.

As an exploratory study in preparation for the main clinical trial, a tDCS intervention in a healthy pediatric population has been designed to target 2 areas of interest: the left Dorsolateral Prefrontal Cortex (lDLPFC) and the right Inferior Frontal Gyrus (rIFG). TDCS is applied to each area of interest both while the participants are performing a task during the stimulation (concurrent task condition) and while the participants are at rest during the stimulation (non-concurrent task condition). Previous studies on tDCS were not conclusive enough about the need to have a concurrent task to increase the response effect of tDCS^25,26^. Therefore, one of the objectives of the exploratory study was to find out whether the response was better with or without a concurrent task. Additionally, three tasks were always performed independently of the stimulation condition (active vs sham, concurrent vs non-concurrent task) and always once the stimulation session has concluded. They were conducted after the stimulation for the sake of evaluating the effect of the stimulation condition in terms of behavioral parameters. Resting state EEG was acquired before and after the tDCS intervention.

We present a methodology for healthy participants’ stratification based on their profile of resting state EEG activity before the tDCS stimulation. The effect of the tDCS intervention on their performance in behavioral tasks performed after the tDCS stimulation is taken into account to label the resulting groups. The considered behavioral metrics correspond to Accuracy and Reaction Times of the Flanker, N-Back, and Continuous Performance Task (CPT) tasks. The performance effect is assessed as the difference in these metrics between the active and the sham conditions. In order to determine the baseline EEG profiles, we extracted spectral features that were found relevant in previous studies in pediatric EEG studies^27^ and in particular ADHD^8,28^. We then computed the relative band power—this measure is preferred over the absolute band power to normalize absolute differences among participants who may present abnormalities in their anatomy^29^. Participant stratification was performed using the algorithms denoted as Spectral Clustering, which has good results for non-convex data^30^, and Fuzzy Clustering, which is robust when boundaries are ambiguous and data contain outliers^31^. A correlational analysis was then applied between the membership of participants to each of the electrophysiologically homogeneous groups and the behavioral assessment metrics after stimulation. Significant correlations of a particular cluster group are tested against the rest of the groups in order to confirm responders. The methodology and results we describe in this article contribute to the understanding of the variability of response to tDCS treatment, with a particular focus on the specific conditions in which the treatment is effective. We support the relationship between the tDCS response and the EEG profiles on the fact that both depend on bioelectrical features of the brain. Hence the EEG features act as a proxy of the biophysical characteristics in each individual’s brain.

Interestingly, the positive responder groups correspond to participants with a non-typical profile of EEG activity, presenting either a slowing of the EEG or a spread of increased alpha rhythm over frontal areas. Our methodology could be applied in the future to stratify patients for tDCS treatment, enabling to focus on those specific digital phenotypes in which tDCS is more likely to become effective. Personalization of treatment, i.e. applying tDCS only to groups of patients predicted to have positive results, would save costs, and time, and improve the efficacy of the treatment at lower sample sizes.

## Materials and methods

### Participants

Data were obtained from a healthy population taking part in a clinical trial for the development of treatments for Attention Deficit Hyperactivity Disorder (ADHD) and Autism Spectrum Disorder (ASD) based on tDCS. The study was approved by the local ethics committee of the Medical Faculty at Kiel University, Germany, and was carried out in accordance with the latest revision of the Declaration of Helsinki and registered in the German Trial Register with number DRKS00008207.

A total of N=56 children and adolescents aged 10–17 years (32 females, mean age: 14.09 years, SD: 2.1) were recruited in the Medical Faculty at Kiel University. Informed consent from the participants and their parents was obtained before the experiment. The exclusion criteria consisted of substance or medication consumption, implants and devices in the body, pregnancy, birth before pregnancy week 37, birth weight lower than 2500 grams, IQ score lower than 80 assessed with CFT 20-R Test (Culture Fair Intelligence Test)^32^, neurological and psychiatric disorders (past or present), brain surgery, presence of epilepsy in the participant or family history, social and health impairments as assessed with the Child Behavior Checklist (CBCL)^33^, the German ADHD Rating scale FBB ADHS^34^ and the Social Responsiveness Scale (SRS)^35^. Participants’ age, sex, IQ and Edinburgh Handedness Inventory (EHI) are given in Tables 2 and 3 of the Supplementary Information.

### Experimental protocol

#### Experimental design

**Figure 1:**
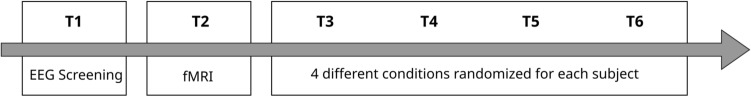
Experimental design. Each participant is assessed at 6 time points, T1 corresponds to an EEG screening, a screening questionnaire and informed consent, T2 to the optional fMRI, and T3-T6 to the randomized interventions (offline/online, tDCS/sham).

A randomized, sham-controlled, double-blind, crossover study design was implemented. Participants came for 6 experimental sessions (see Fig. 1). The first 2 sessions (T1-T2) correspond to participants screening and an optional fMRI scan. Participants then came back 4 times to receive the tDCS interventions (T3-T6) which were of 4 different types, administered in a randomized order: active tDCS with a concurrent task, active tDCS with a non-concurrent task, sham tDCS with a concurrent task and sham tDCS with a non-concurrent task. Additionally to this experimental condition, which includes the concurrent task in the concurrent-task conditions and no task in the non-concurrent task conditions, 3 tasks were included in the experimental design for behavioral effect evaluation. These 3 tasks were conducted after the stimulation finished for the sake of evaluating the effect of active or sham stimulation in terms of behavioral parameters. These 3 tasks were always performed independently of the stimulation condition (active vs. sham, concurrent vs. non-concurrent task) and always once the stimulation session had concluded. There was a minimum washout period of 7 days in-between sessions. At the start of each stimulation session, participants were asked to complete an assessment of their current mood, motivation, and any adverse event since the last session. Following the stimulation session, participants completed a questionnaire on the safety, tolerability, and blinding of the stimulation.

**Figure 2:**
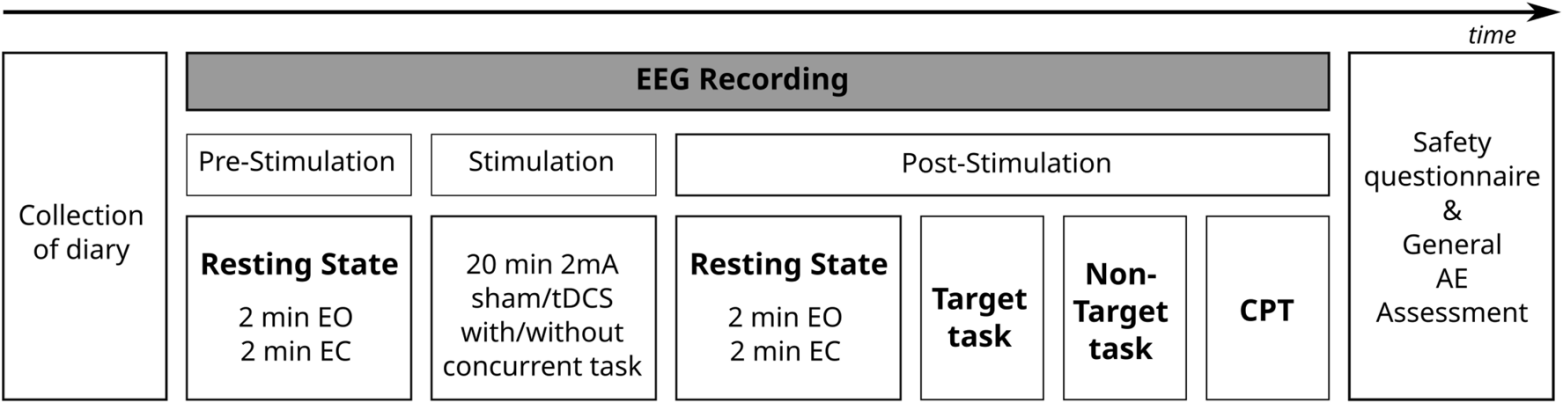
T3 to T6 trial design. During each trial, 2 min eyes open and 2 min eyes closed resting state EEG are being recorded before and after the tDCS stimulation. Participants then came back 4 times to receive 4 different tDCS interventions (T3–T6) in a randomized order: active tDCS with a concurrent task, active tDCS with a non-concurrent task, sham tDCS with a concurrent task and sham tDCS with a non-concurrent task. After each tDCS intervention, 3 tasks were included in the experimental design for behavioral effect evaluation. These 3 tasks were always performed after the stimulation session had ended, and independently of the stimulation condition (active vs. sham, concurrent vs. non-concurrent task). Each stimulation session lasted 20 min. For the concurrent type of intervention, the task started and ended 2.5 min after and before the stimulation respectively (in order to avoid current ramping distractions). The tasks performed after the stimulation had a duration of approximately 16 min for Flanker Task and N-Back N-Back Task, and approximately 18 min for CPT.

Participants were randomly divided into 2 groups: participants in Group A were stimulated in the left Dorsolateral Prefrontal Cortex (lDLPFC) and performed the N-Back Task in the concurrent task condition, whereas participants in Group B were stimulated in the right Inferior Frontal Gyrus (rIFG) and performed the Flanker task in the concurrent task condition. In each stimulation session, both groups randomly received either a sham or an active tDCS stimulation. Each tDCS stimulation session unfolded as follows: once participants were equipped with the stimulation headset, resting state EEG was recorded during 2 min eyes open (EO) and 2 min eyes closed (EC), followed by 20 min of tDCS stimulation. In the concurrent task condition, the task started after 2.5 min of tDCS stimulation and ended 2.5 min before the end of the stimulation. In the non-concurrent task condition, participants were instructed to sit and relax with eyes opened while receiving tDCS. For all participants, after stimulation, EEG data were recorded during a second resting state period (2 min EO, 2 min EC) and then during the execution of the following cognitive tasks: Flanker Task, N-Back Task and Continuous Performance Task (Fig. 2).

#### Transcranial direct current stimulation

**Figure 3:**
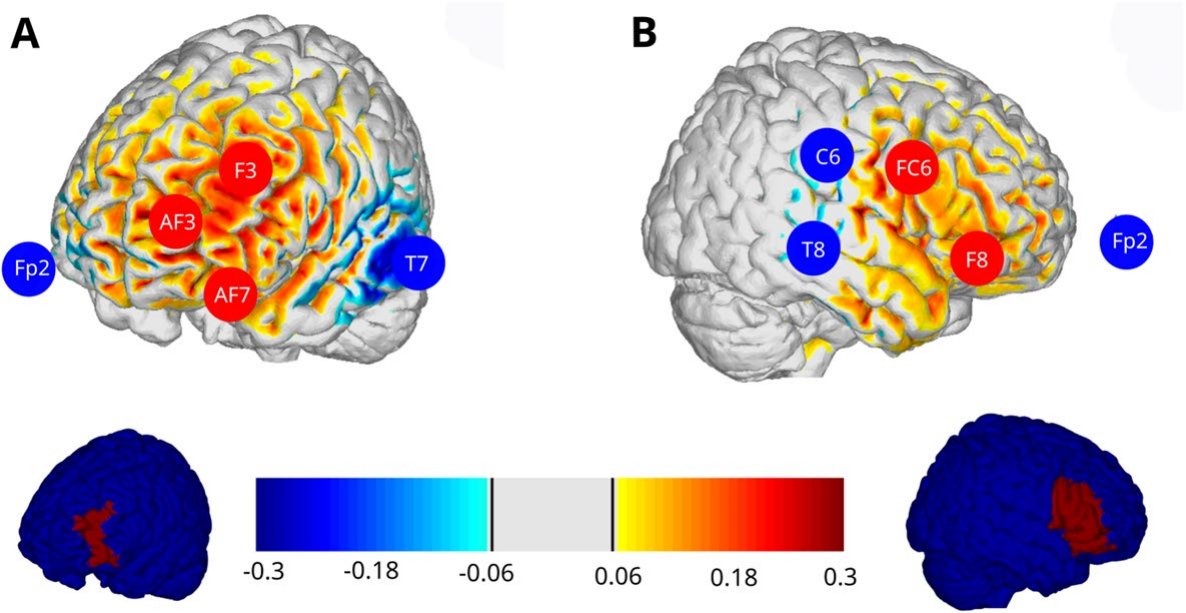
Stimulation areas. Brain renderings showing the component of the E-field normal to the cortical surface (En), positive/negative values indicate the into/out-of the cortical surface direction of the En, leading to an increase/decrease of excitability in cortical pyramidal cells. Group A of participants received stimulation in (**A**) left Dorsolateral Prefrontal cortex (see target area in the bottom row A). Group B of participants received stimulation in (**B**) right Inferior frontal gyrus, the montage was optimized defining a target of Broadmann-areas 44, 45 and 47 on the right hemisphere (see target area in the bottom row B).

Transcranial direct current stimulation (tDCS) was applied using a Starstim 32 multi-channel stimulation device (Neuroelectrics - Barcelona, Spain). The montage consisted of 5 circular electrodes (3.14 cm²) filled with gel. The total stimulation current consisted of anodal 2mA during 20 min, with a 30 s of ramp up and down at the beginning and end of the stimulation (see Fig. 3). The interested reader can find details on the DLPFC^36^ and the IFG^37^ montages in the referred works.

#### EEG recording

EEG data were recorded using the same device used for the stimulation at a sampling rate of 500 Hz with 32 channels, following the 10-20 system: P8, T8, AF7, AF8, F8, F4, C4, P4, FC5, AF4, Fp2, Fp1, AF3, Fz, C6, Cz, C5, PO3, O1, Oz, O2, PO4, Pz, Fpz, FC6, P3, C3, F3, F7, FCz, T7, P7.

#### Behavioral tasks

**Figure 4:**
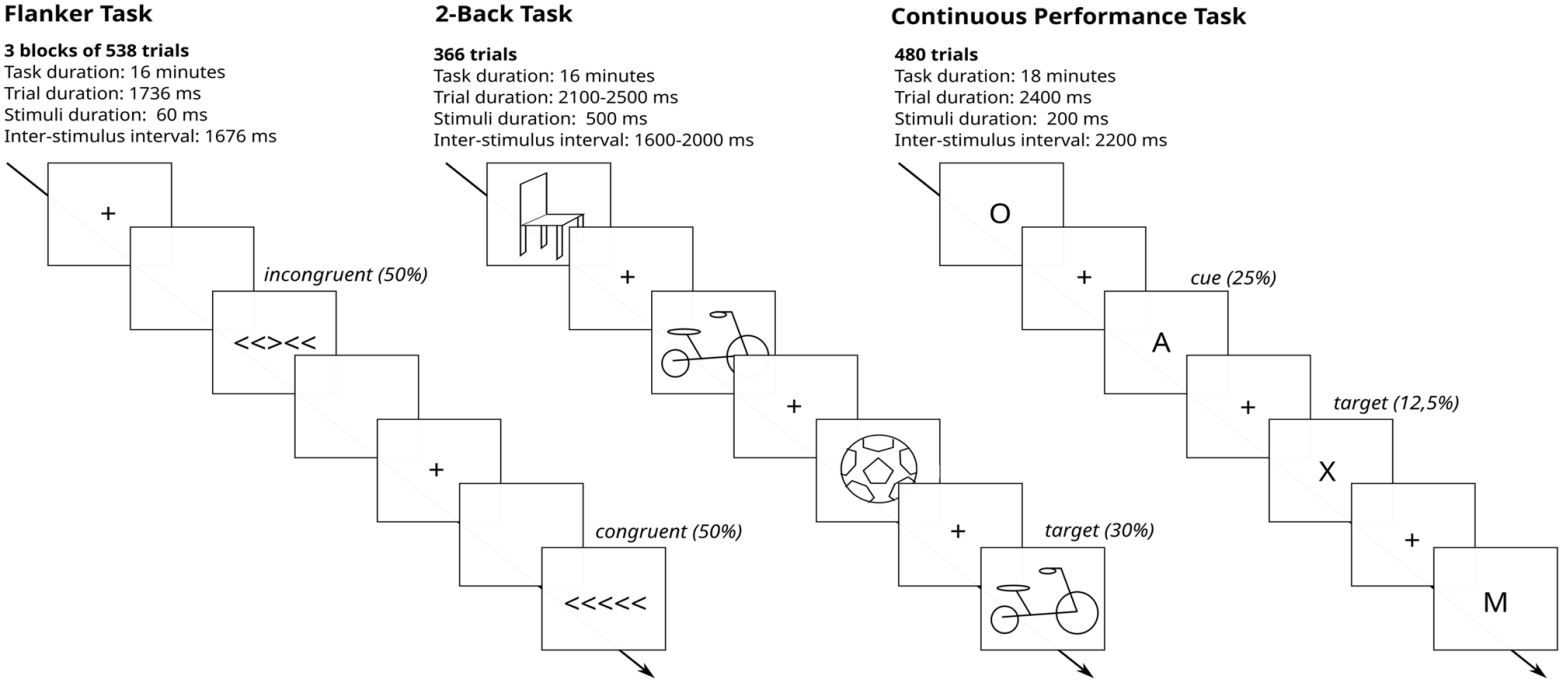
Behavioral Tasks. Examples for Flanker Task (left), 2-Back Task (middle) and Continuous Performance Task (right). In the Flanker Task^38^, 5 arrows in a row were shown in which the middle one (target) points to the same or opposite direction as the rest (distractors). Participants had to indicate if the target pointed left or right. During the 2-Back Task^39^ participants had to detect if a presented picture is the same as a picture shown 2 steps back. In the Continuous Performance Task^40^, participants were presented with a series of letters. They were shown pairs of letters in which a cue is followed by a target. They had to respond when the target appears, only when it is preceded by the cue (which is only one of the 4 existent possibilities).

Behavioral tasks included the Flanker Task, N-Back Task and Continuous Performance Test (Fig. 4). These were programmed using Presentation® Software (Version 20.0, Neurobehavioral Systems, Inc., Berkeley, USA). Behavioral performance metrics were computed in R (version 3.6.1, R Core Team). The primary endpoints are the N-Back Accuracy for participants in Group A and the Flanker Accuracy for participants in Group B. For more details please see the associated experimental work^36^.

### EEG signal processing and feature extraction

The EEG data analysed here correspond to the 2-min resting state EEG in eyes closed condition recorded before the start of tDCS stimulation in each T3 to T6 trial for each participant. Data were first band pass filtered using a 1000 coefficients FIR filter with cut-off frequencies at 2Hz and 45 Hz, demeaned and detrended. Frontal channels AF4, Fp2, Fp1 and AF3 were removed from further processing and analysis due to the presence of numerous eye-movement related artifacts. High amplitude artifacts were removed using an amplitude threshold of 100 μV. Finally, the data were re-referenced to electrode Cz, which was then excluded from further processing. The relative band power was computed in 4-s time-windows epochs with 50% overlap in bands delta (2–4 Hz), theta (4–8 Hz), alpha (8–13 Hz), beta (13–30 Hz) and gamma (30–45 Hz). The final relative band power values for each subject and session were taken as the median of the epochs. The EEG data pre-processing was done in Python 3.9 using custom scripts and Python packages (i.e. MNE).

### Overview of the analytical approach

**Figure 5:**
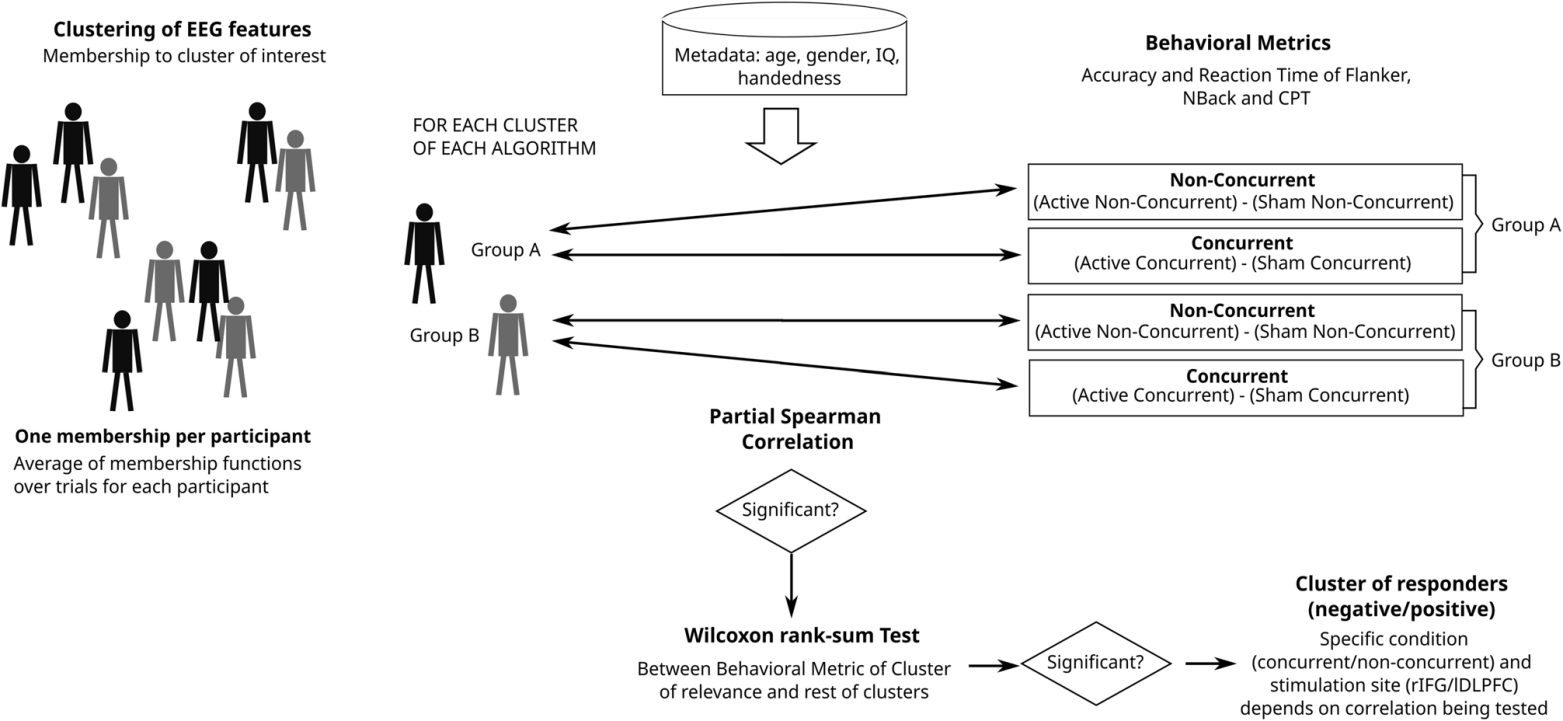
Schematic of clustering and Wilcoxon test pipeline. EEG features are clustered and membership values are correlated with different behavioral metrics. If significance is reached (correlation *p*-value is less than 0.05), a Wilcoxon Test is done between the behavioral metric of that cluster and the rest of the points.

Figure 5 gives a schematic of the methods used in the core analytical approach of our work. Details of each step are given in the following Sections. All analysis stages were implemented in Python 3.9 using Scikit-learn and SciPy packages.

### Unsupervised clustering analysis

We applied 2 different unsupervised clustering algorithms to the spectral features extracted from the EEG data: Spectral Clustering^30^ (implementation from Python scikit-learn package^41^) and Fuzzy C-Means^42^ (implementation from Holt Skinner 2018^43^). The definition of parameters for the clustering pipeline consisted of selecting the optimal number of clusters *k* ranging from 2 to 6, and the specific parameters for each algorithm (number of components *n* for Spectral Clustering and fuzzification parameter value *m* for Fuzzy C-Means). The parameters were chosen based on several internal validity measures and the visual inspection of the feature space in a 2-dimensional projection based on the t-distributed stochastic neighbour embedding (t-SNE). The used internal validity metrics are: Silhouette, Davies Bouldin Score (DB), Calinski-Harabasz (CH), Bayesian Information Criteria (BIC) and Inertia (or Within-Cluster Sums of Squares). Homogeneous clustering is represented by low values of DB, Inertia and BIC, and high values of Silhouette and CH. We experienced a relative lack robustness of these metrics in our large dimensional space (5 different band power values for each of the 27 EEG channels). Therefore we added a second criteria for the selection of the parameters, namely the t-distributed stochastic neighbor embedding (tSNE) visualization. TSNE is appropriate for maintaining local interactions, addressing non-linearity and robust to the presence of outliers^44^, and showed to be flexible enough to visually depict in a very clear manner the clusters determined by the employed algorithms. We have also used PCA for visualization and found tSNE was offering a better visualization in terms of interpretability. This may be due to PCA considering linear interactions, and not being robust to the presence of outliers that spuriously increase the variance of some dimensions.

#### Cluster membership definition and computation

For each participant we used 4 independent recordings (corresponding to each pre-stimulation EEG resting state recorded in sessions T3–T6). We thus obtained 4 different membership functions for each subject, which may present small variability due to the time between recording sessions, i.e. 1 week. The FCM algorithm directly provides cluster prototypes and a fuzzy membership function of each particular datapoint to the different clusters. In Spectral Clustering, which does not provide these as direct outputs, cluster prototypes are first calculated as the average of the points belonging to each specific cluster. The membership is then calculated as a probability function of the Euclidean distance from each datapoint to the aforementioned prototype. As we were trying to establish a participant profile, we averaged the cluster membership functions of each subject over the 4 sessions. This average membership function is expected to be more robust than the individual session membership and to get rid of the temporal variability of subject profiles.

It is worth pointing out that the membership of each data sample to each of the clusters is defined in a real-valued domain in order to avoid loosing information due to the binarization of the data. By defining memberships in a real-valued domain we are able to treat differently the points that are close to the cluster centres and the points that are far from them, i.e. since they present very different membership values to the cluster under consideration. This is expected to increase the robustness of the analysis pipeline, given the fact that early binarization thresholds largely influence final results.

### Characterizing digital EEG phenotypes through statistical analysis

#### Correlation between clusters and behavioral data

After clustering, we correlated the membership function and the behavioral data of the tasks after tDCS of each participant using a Partial Spearman Correlation. This type of correlation allows to take into account spurious factors that may affect the EEG features. The correlation was corrected for age, sex, IQ and handedness. The False Discovery Rate (FDR) multiple comparisons correction was applied in order to avoid random significant values of the correlation measure using the Benjamini and Hochberg step-up procedure (BH)^45^.

The purpose of the correlational analysis is to label the clusters of homogeneous EEG digital phenotypes that we obtained either as positive, negative or non-responders to the tDCS intervention depending on their behavioral response. As behavioral response we used the accuracy and reaction times in the Flanker task, N-Back and CPT tasks performed after the tDCS stimulation. In order to obtain a unique behavioural measure to conduct the correlation, we computed the difference in performance between the active and sham tDCS conditions for each task and behavioural metric (accuracy and reaction time). This performance difference was computed independently for trials with a concurrent task during tDCS and trials without a concurrent task.

From combining the 2 stimulation sites (Groups A/B) with the 2 task concurrency conditions (concurrent/non-concurrent) we obtained 4 different experimental conditions and thus for each clustering algorithm 4 correlations were computed. Hence we correlated the performance difference between active and sham tDCS of each experimental condition and the average membership to the cluster of each subject. The rationale for computing these 4 correlations is to identify the concrete condition, i.e. stimulation site A/B and task concurrence, in which significant results are found and predict later the response of the subjects who fall into the same cluster. If there is a significant correlation (results are considered statistically significant if the corrected correlation p-value is less than 0.05), we can establish a relationship between the digital EEG profile and the behavioral performance difference between the active and sham conditions. Clusters of positive responders are defined as showing positive accuracy correlations and negative RT correlations, i.e. positive responders show higher accuracy and smaller RTs following active tDCS than following sham tDCS. On the contrary, clusters of negative responders, are defined as presenting a negative correlation with Accuracy and positive correlation with RT measures.

#### Statistical comparison of behavioral measures between clusters of positive and negative responders using the Wilcoxon Test

This step aims at testing the significance of the difference in behavioral response between each specific clusters of positive or negative responders. If any of the 4 correlations computed for each cluster showed significance (positive or negative), we then analysed the difference in behavioral response between the specific cluster members and all non-members of the cluster. Hence, a rank-sum Wilcoxon Test was calculated on the behavioral metrics within each cluster samples and the samples belonging to the remaining clusters. The FDR Benjamini and Hochberg step-up procedure^45^ was used here again to correct for multiple comparisons.

In this part of the analysis we want to assess if there are significant differences between the behavioral outcomes for each specific cluster, once the cluster has shown a correlation between the membership to that cluster and the corresponding behavioral metric. We illustrate this operation with an example: If a significant correlation is found for Cluster 1 in behavioral metric Accuracy of the Flanker task—i.e. considering the membership values of all data-points to that specific Cluster 1—then a Wilcoxon test is performed on the Flanker Accuracy active-sham difference. In this test we compare the performance of the subjects who belong to Cluster 1 versus the rest of the subjects. The objective of this step is to have an estimation of the improvement in the treatment response in case the clustering analysis is used for recruiting subjects in a future clinical trial.

At this point, since we have been calculating the significant difference between behavioral response of the subject samples belonging to a cluster and the rest of the subject samples, we need to determine to which final cluster a given subject belongs to. This was done using the $\arg \max$ function over the membership values to the different clusters, i.e. determining the cluster with a highest membership value (e.g., if point X has a membership value of 0.2, 0.3, 0.4 and 0.1 for Clusters 1, 2, 3 and 4 respectively, then it would belong to Cluster 3).

## Results

### Feature space analysis

**Figure 6:**
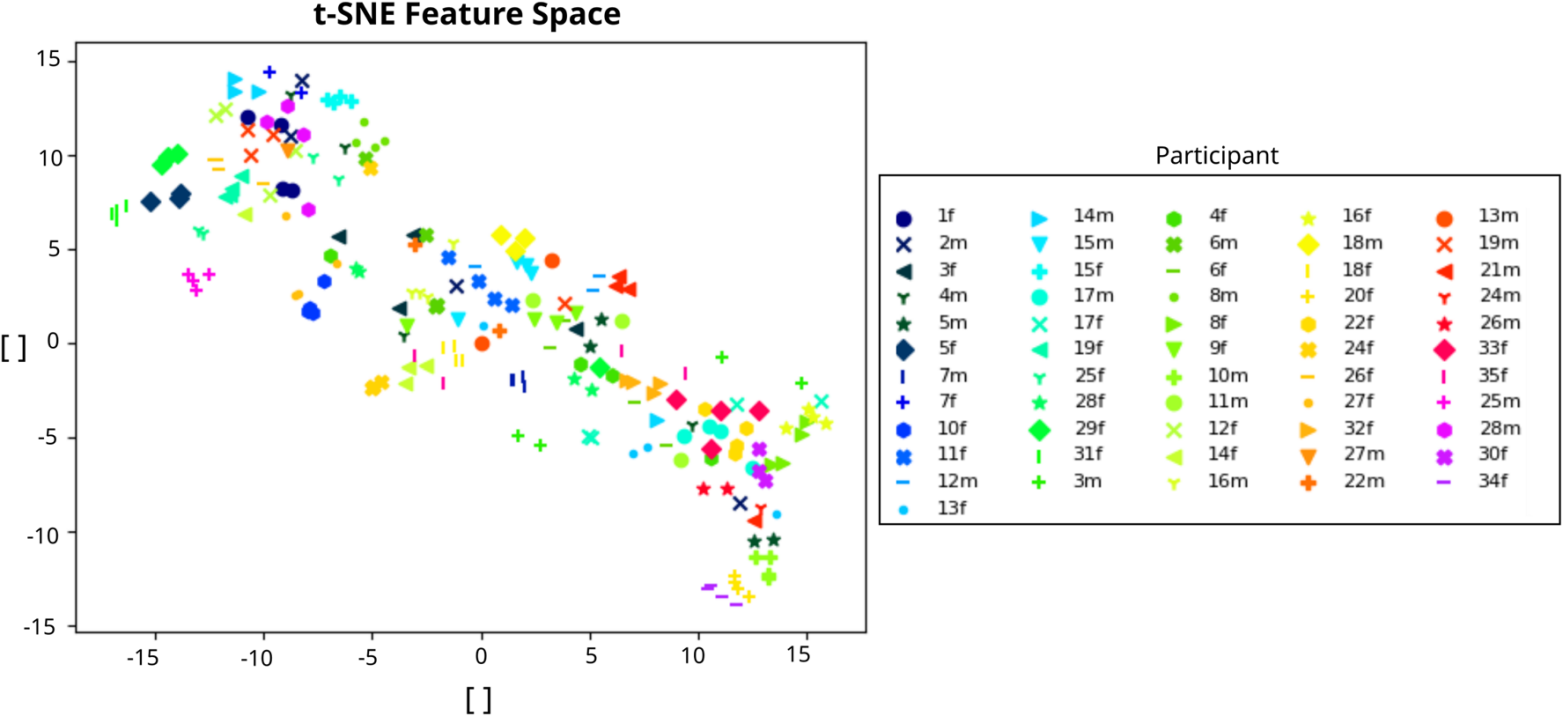
Feature space. Visualization of EEG data-points with t-SNE in 2 dimensions. Each point corresponds to a specific trial for each participant, the features of each data-point are the RBP in each channel.

We computed the relative band power in the delta, theta, alpha, beta and gamma frequency bands for each electrode, resulting in a feature space of 135 components. After removal of any artifacts and due to missing trials or dropout subjects, 206 data points were used in the analysis. Figure 6 shows the feature space visualization with t-SNE in 2 dimensions, given by an optimal perplexity index value of 30^44^. One interesting aspect here is to observe how reproducible is our approach given that for each subject there was a 1-week delay between the recording of each of the 4 samples. We can observe that the samples of all 4 trials for each participant are very close to each other. Therefore we can state that the intra-subject variability is small enough to consider the feature representation as a robust one, which gives grounds for averaging the membership functions of a participant’s 4 trials in order to obtain a unique membership per subject for the correlational analysis.

#### Clustering results

**Figure 7:**
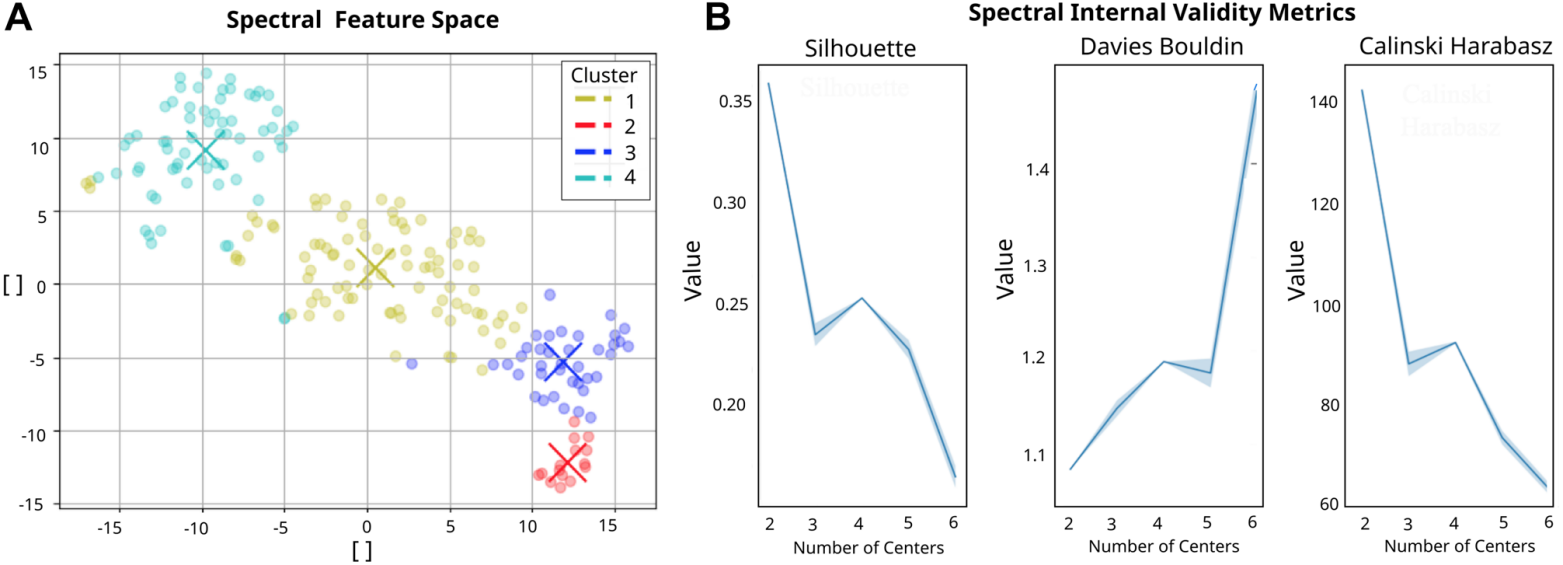
Clustering results for spectral clustering. The left plot shows the feature Space, the colour code represents each cluster. From all data-points, 42,7% lie in Cluster 1, 7.3% in Cluster 2, 18.4% in Cluster 3 and 31.6% in Cluster 4. The right plots show the evolution of the internal validity metrics Silhouette and Davies Bouldin when changing the number of centres. An elbow at k=4 can be seen.

We implemented the different clustering algorithms to establish homogeneous subgroups among subjects based on their resting-state EEG spectral features before intervention. The evolution of the validity measures over the different parameters does not offer a clear criterion for parameter selection. We believe this may be due to the high dimensionality of the feature space, i.e. the “curse of dimensionality”^46^, which seems to be not clearly tackled by the most common validity measures. Nevertheless, we observe some singular points in the evolution of the selected validity measures over clustering parameters (see Fig. 7B). Given the shortcoming of the validity measures to offer clear objective criteria for parameter selection, we have taken into account an additional qualitative evaluation criteria based on the visualization of the t-SNE projection of the clustered feature space. Figure 7 A shows well separated and clear cluster clouds. For the Spectral Clustering, a number of components *n* equal to the number of centres was used, as it generally corresponds to the default parameter. This gives better results than a number of components *n* equal to 100 applied as an example for extreme values, and gives similar results than small values such as 2, 6 and 8. For FCM the chosen parameter was a fuzzification parameter *m* of 1.7 since it differs sufficiently from 1 (so as to be more dissimilar to K-means) and presenting optimal values for the validity metrics and visualization in the feature space.

Concretely we can observe some singular points, i.e. “elbows”, for $k=4$ clusters for the Spectral Clustering algorithm that correspond to the optimal values. For the case of the FCM algorithm it is optimal in $k=5$. In order to have the largest amount of data-points in each cluster cloud, and since it shows clearly defined clusters in the visualization from both algorithms, we eventually defined the number of cluster centres as $k=4$. Since the clustering output visualized in the feature space shows similar results for both algorithms, a possible hypothesis for the light differences in point cluster assignment between Spectral and FCM is the difference in the way the membership functions are computed in each algorithm (FCM results for $k=4$ can be found in Supplementary Information for the sake of comparison). Given the similarity of the clustering results the results in the following sections are shown only for the Spectral Clustering.

#### Clustering validation

The robustness of the parameter search in the clustering methodology was validated through an M-hold-out using 80% of the data for parameter search. The chosen algorithms with their corresponding parameters (i.e. fuzzification parameter *m* of 1.7 for FCM and a number of components *n* equal to the number of centers for Spectral Clustering) and number of centers *k* were found out in a subset of the data points and then tested in the remaining points, checking whether the clusters assigned in the test set generalize from the ones obtained on the training set. This was done by first clustering the train data samples and plotting them in the feature space. The remaining test data sample labels were then assigned according to their nearest distance to the final center obtained previously.

The validation is successful based on the visual overlap between the clusters assigned to the test set, which are not used in the parameter search (see Figure 6A in Supplementary Information). Moreover, the internal validity metrics follow the same trend in both data subsets (see Figure 6B in Supplementary Information).

#### Digital electroencephalography phenotypes

**Figure 8:**
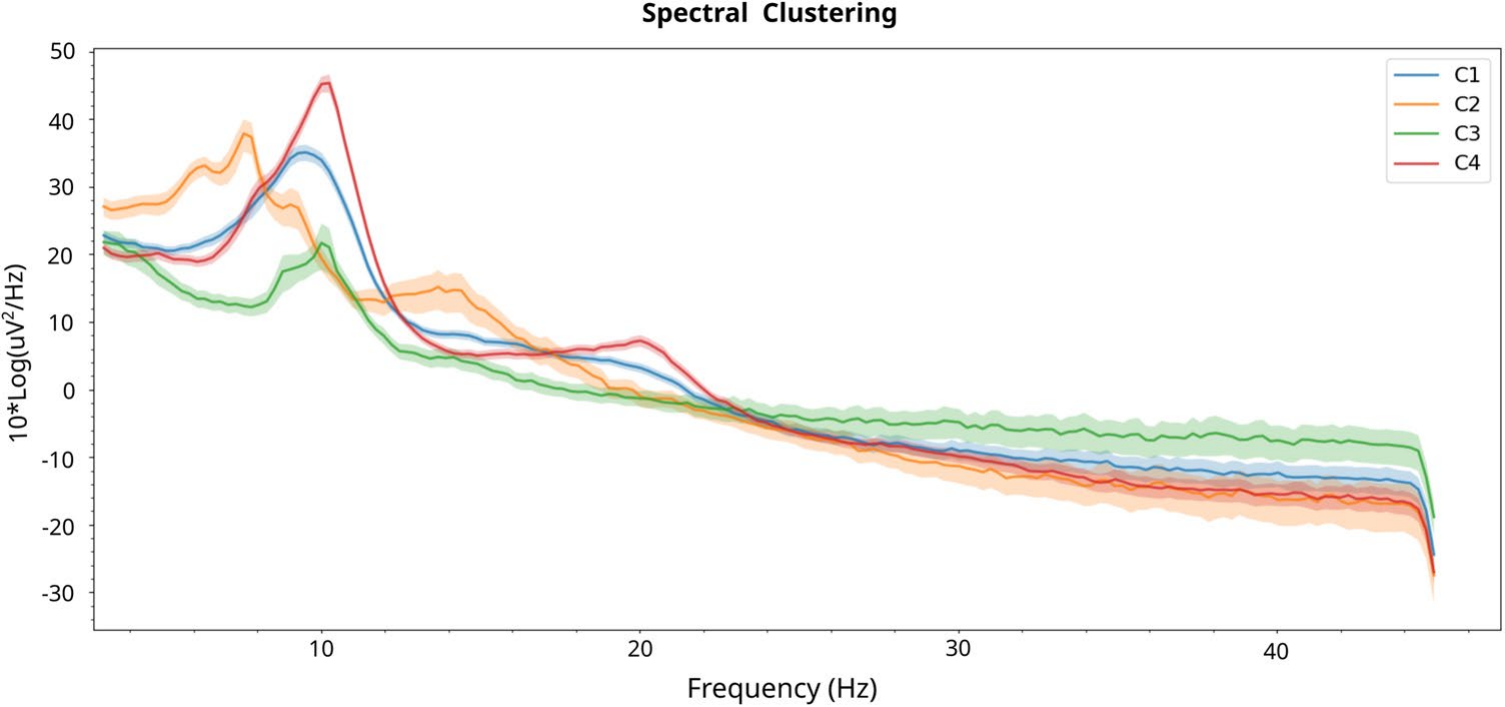
Power spectrum density of each cluster for spectral clustering results.

**Figure 9:**
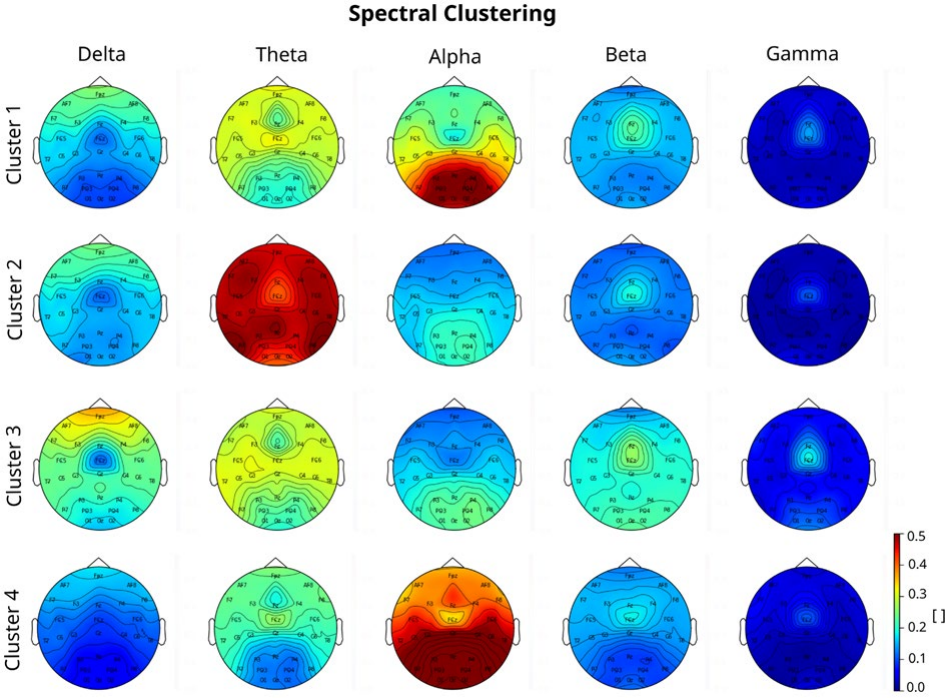
Power spectrum plot of each cluster for spectral algorithm results.

In order to characterize each of the clusters in terms of the associated digital EEG profiles, the prototypes generated for each cluster of each algorithm were used. The electrophysiological profiles are set up as a combination of a representation in the frequency and spatial domains. First, the power spectral density (PSD) for each cluster prototype is plotted (Fig. 8) using the mean of occipital and parietal electrodes (i.e. O1, O2, Oz, P8, P4, P3, P7, PO3, PO4, Pz). Taking the alpha peak during eyes closed as a reference, differences can be observed between the different clusters. These can be further analyzed on hand of the scalp power distributions in the spatial domain. Topoplots are used for the representation of prototypes in the spatial domain (Fig. 9). They are visualized by calculating the mean of the relative band power of all of the data points belonging to each cluster.

**Figure 10:**
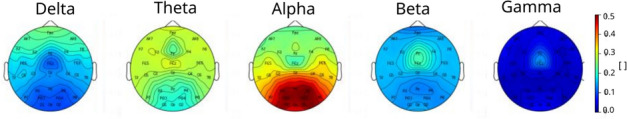
Overall population topographical map.

Cluster 1 presents the neurotypical scalp power distribution for eyes closed, with alpha power localized over posterior regions. This could be observed as well in the PSDs (see Fig. 8). Cluster 2 presents a very clear slowing in both the PSD figure and the topographical maps, from the alpha band towards the theta band. Cluster 3 presents low amplitude at 10 Hz and large amplitudes at higher power values in the PSD. Cluster 4 presents the largest amplitude at 10 Hz and a peak at 20 Hz in the PSD, and the difference between frontal and parietal areas for the alpha band in the topoplot is smaller compared to the rest of the clusters. As a remark, Cluster 1 topographical map of activation is the most similar to the ones of the overall study population (see Fig. 10).

**Figure 11:**
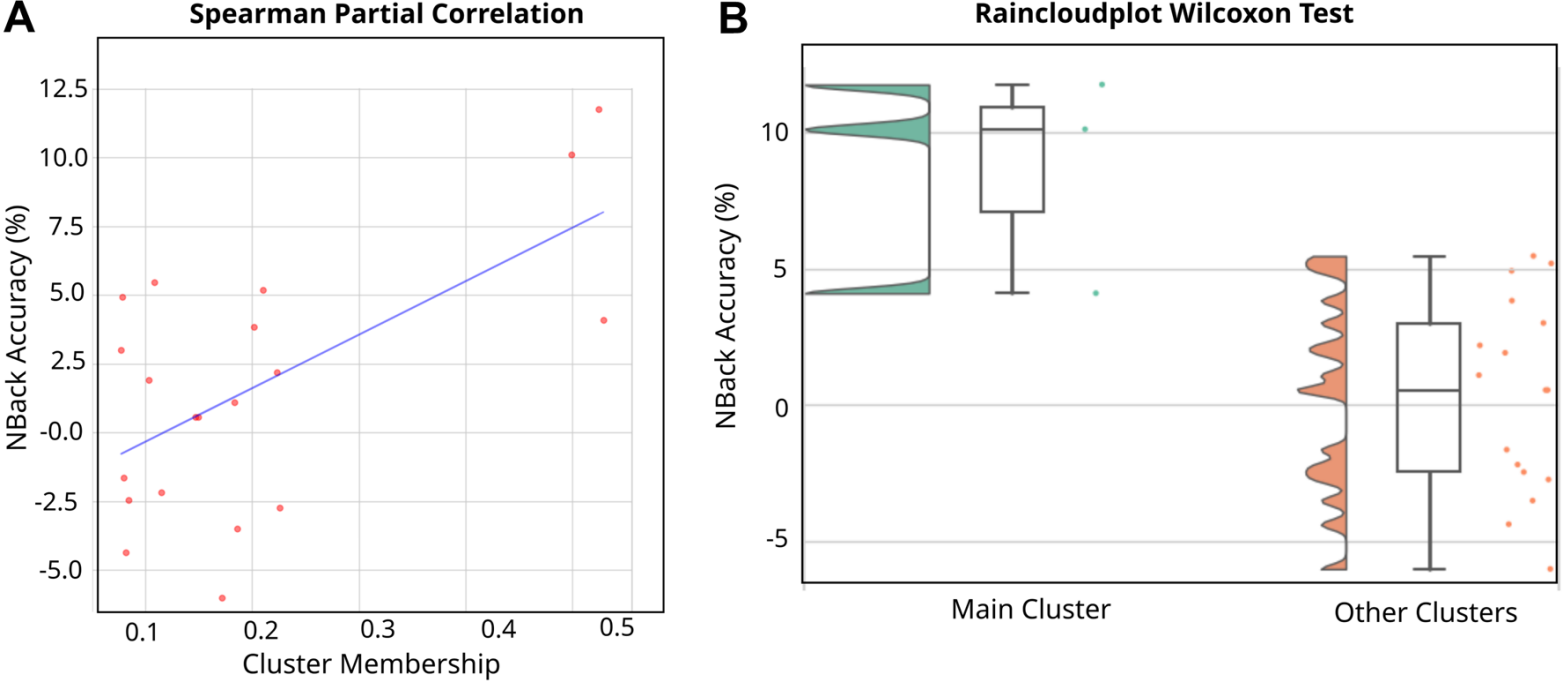
Example of significant positive responder group for Spectral Clustering (Cluster 2), with rIFG stimulation with no concurrent task, measured with N-Back Accuracy. (**A**) Correlation between cluster membership of pre treatment EEG spectral features of all participants and N-Back Accuracy at the end of the trials. Best-fit line showing least square polynomial fit. (**B**) Wilcoxon Test between N-Back Accuracy of Cluster 2 Spectral members, and the members of the rest of the Clusters. The raw data is visualized as points as well as the probability density, and key summary statistics in the boxplot (median, mean, and confidence intervals).

### Behavioral correlational analysis

We applied the described correlation analysis between participants’ membership to each cluster and the post-tDCS behavioral results of Accuracy and RT for the Flanker, N-Back and CPT tasks. Due to the low variability in the baseline EEG spectral activity of each participant (Fig. 6), the average of the membership function over all trials of each participant was computed as formerly mentioned. Eleven subjects were excluded due to incorrect randomization and/or dropouts during the trials, and behavioral outliers are removed for each metric using a threshold of $+$/$-2.5$ standard deviations. The correlational analysis can be used to label the clusters in terms of their positive or negative responding nature. A specific example of the positive response is that of cluster 2, whose members are positive responding to the rIFG stimulation with non-concurrent task (see Fig. 11). Negative responses are detected by the correlational analysis as well (see Figure 5A in Supplementary Information).

**Figure 12:**
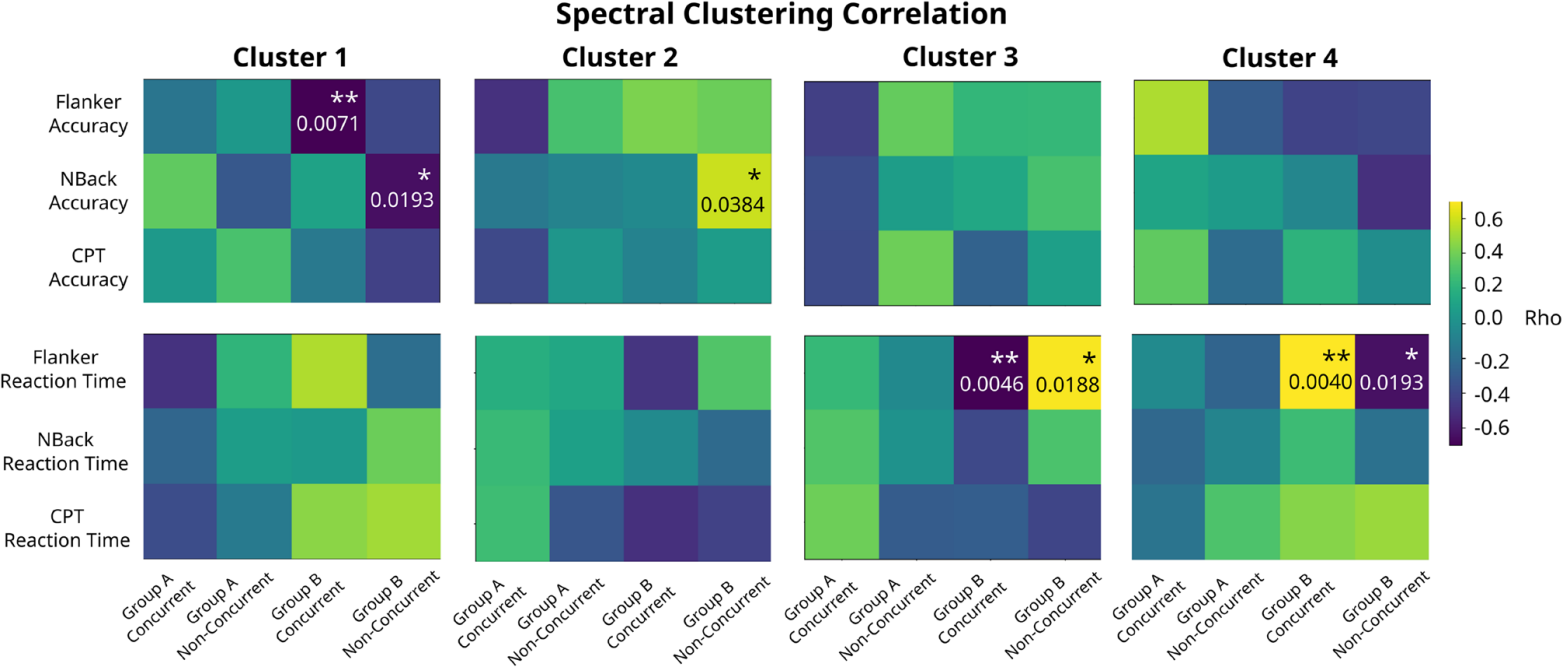
Correlation results for spectral clustering. Each matrix row corresponds to a specific behavioral task endpoint, and each matrix column corresponds to a particular treatment protocol. The colour bar represents the sign of the correlation (positive or negative) and *p*-values are written in the significant cells. For Group B, 24 corrections are done; for Group A, 20 corrections are applied.

The following matrices (Fig. 12) show the strength of the partial Spearman correlation and the range of the *p*-values when significant ($$^{*} - 10\%, ^{**} - 5\%$$ and $$^{***} - 1\%$$) after correction for multiple comparison with FDR (24 corrections for Group B, 20 corrections for Group A). These matrices summarize the findings achieved by the correlational analysis.

### Differences between cluster versus non-cluster members

We computed the rank-sum Wilcoxon test only in clusters with significant correlation results. Responder clusters (positive or negative) are analyzed with respect to the difference to non-responder ones. Responding clusters are confirmed by giving significant differences in this test as well (see Fig. 11 for an example of a positive responder cluster and Figure 5B in Supplementary Information for a negative one). Corrected results for multiple comparisons for the Wilcoxon analysis, as well as number of corrections, are presented in Table 1.

The robustness of the correlational analysis was tested through a 1000-hold-out validation with different percentages of the data set (specifically 80%, 60% and 40%). We find a trend for the coefficients and *p*-values of both correlations and Wilcoxon tests (see example in Supplementary Figure 7). Overall, the subsets have the same direction of correlation as the whole dataset, and the strength goes diminishing in the sequence 80%–60%–40%. With respect to the correlation *p*-value, it is almost always significant (correlation *p*-value is less than 0.05) for the 80% dataset. In the 60% dataset it is generally non-significant although very close to the boundary (correlation *p*-value near 0.05) and for the 40% it is never significant. In both cases, the standard deviation increases when the percentage of data-points decreases. These takeaways have similar trends for the analysis using Wilcoxon test. For the case of the distance to the original fit line (with the 100% of data-points), the mean is very similar in all percentages of the dataset, whereas the standard deviation increases for decreasing percentages. Overall, results show that our correlation method is robust and that the sample size used is the minimum required for obtaining these results.

**Table 1:** Spectral significant responders.

Cluster	Group A, Concurrent	Group A, Non-concurrent	Group B, Concurrent	Group B, Non-concurrent
1			Flanker accuracy (−, 0.0150)	
2				N-back accuracy (+, 0.0345)
3			Flanker RT (+, 0.0150)	Flanker RT (−, 0.0433)
4			Flanker RT (−, 0.0402)	Flanker RT (+, 0.0345)

These results correspond to significant correlations and significant Wilcoxon tests. In parenthesis are: (1) type of response (positive or negative), (2) Wilcoxon *p*-values. There are significant correlations only in Group B, therefore for Wilcoxon Test 4 corrections are done for Non-concurrent Group and 3 for Concurrent Group. Stimulation protocol Group B (rIFG), offline mode (non-concurrent task) presents positive response in Clusters 2 and 4 measured with N-Back Accuracy and Flanker RT respectively, and negative response in Cluster 3 assessed with Flanker RT. Stimulation protocol B (rIFG), active mode (with Flanker concurrent task) presents negative response in Clusters 1 and 4 measured with Flanker Accuracy and Flanker RT respectively, and positive response in Cluster 3 assessed with Flanker RT.

**Figure 13:**
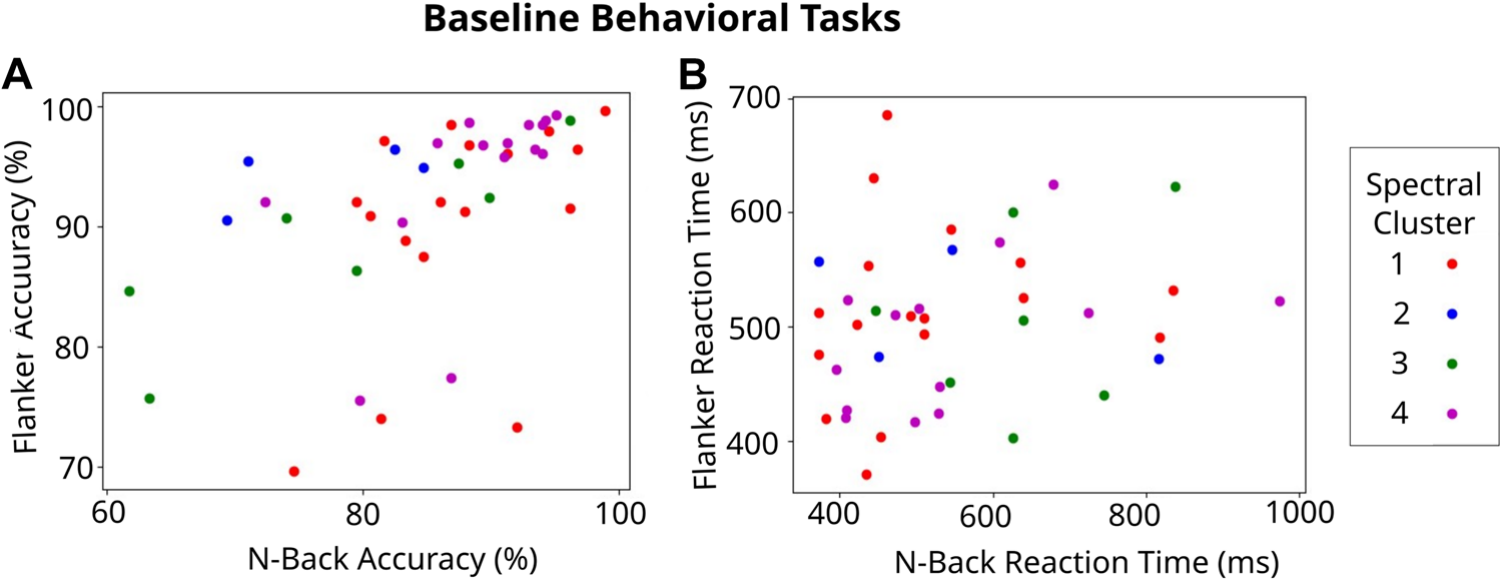
Baseline behavioral tasks distinguished by Spectral Algorithm clustering for (**A**) accuracy and (**B**) reaction time.

Since previous work state that subjects who better respond to treatment correspond to those who start in worst conditions^26,47,48^, an extra analysis was done in order to discard any effect of this factor on the observed differential response. Hence we further studied whether there is a relationship between baseline behavioral performance (as measured in the first session at T3) and treatment response. The following Fig. 13 shows the behavioral response for the baseline (T3) of the main endpoints (Flanker Accuracy and N-Back Accuracy) as well as for the RT of those tasks. No cluster structure can be seen on the behavioral metric at baseline. Therefore we can state that the cluster structure is solely due to homogeneous digital electrophysiological profiles and not due to a differential baseline response to the tasks.

## Discussion

Our work provides a novel approach to study the effects of multichannel anodal tDCS treatment in healthy children and adolescents with different specific brain profiles. This is of interest given the limitations of existent treatments in pediatric clinical populations, e.g. ADHD and ASD, for which new treatments with no side effects^49^ are being developed. The unsupervised learning approach we propose constitutes a good alternative to the use of one-model-fit-all approaches based on supervised learning, whose issues to predict behavioral response have been recently analyzed^50^. Furthermore the prognostic value of unsupervised approaches can be exploited in the context of the development of reliable and well-defined personalized protocols. Given that a significant positive response to treatment is not always guaranteed, the proposed subject stratification approach can be of enormous value for the cost-effective development of new treatments.

**Figure 14:**
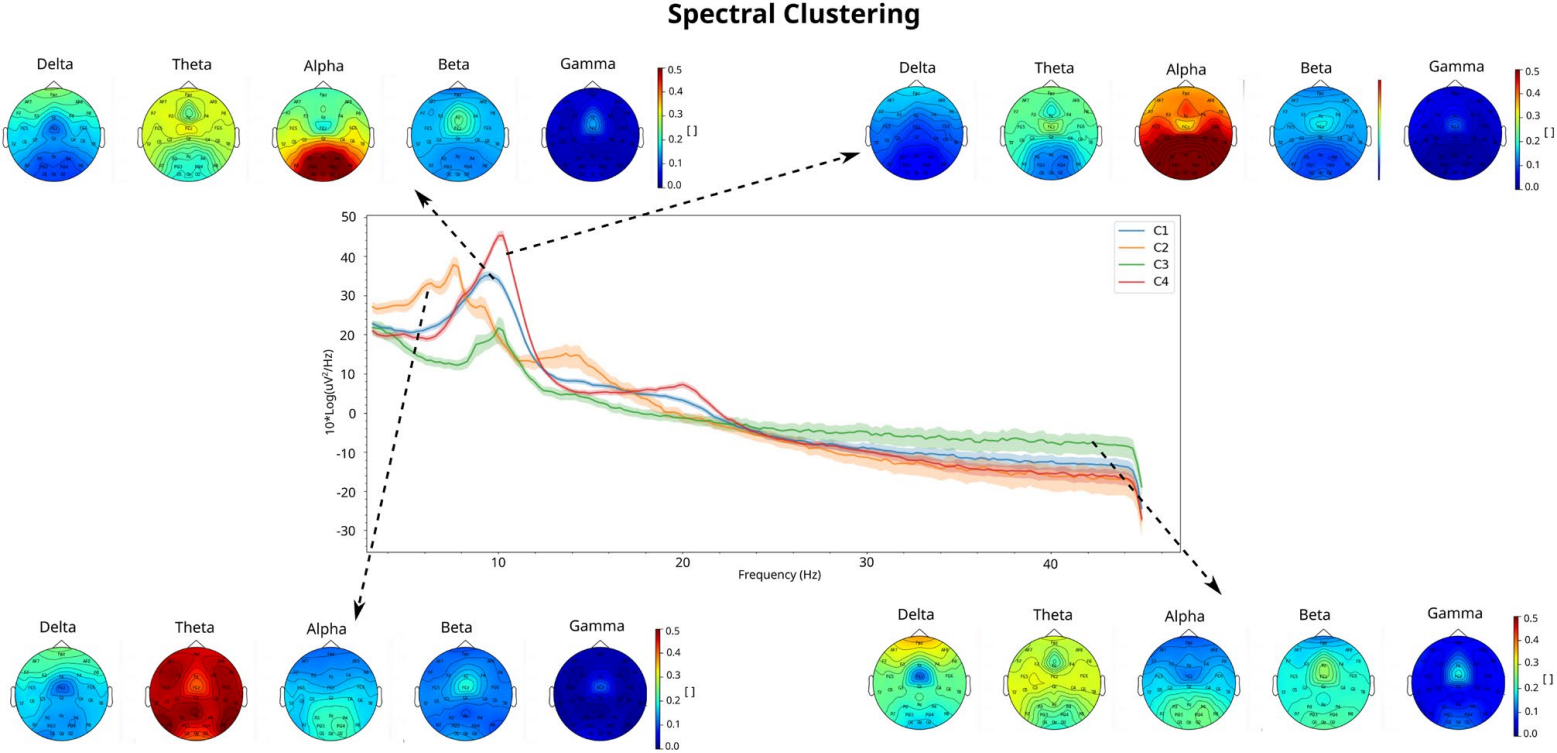
Summary of EEG prototypes. Topoplots and PSD for Clusters that present a significant response in Spectral Algorithm. Cluster 1 (blue PSD) has a negative response to rIFG stimulation with Flanker concurrent task, measured with Flanker Accuracy. Cluster 2 (orange PSD) has a positive response to rIFG stimulation with no concurrent task, measured with N-Back Accuracy. Cluster 3 (green PSD) has a positive response to rIFG stimulation with Flanker concurrent task, measured with Flanker RT; and a negative response to rIFG stimulation with no concurrent task, measured with Flanker RT. Cluster 4 (red PSD) has a positive response to rIFG stimulation with no concurrent task, measured with Flanker RT; and a negative response to rIFG stimulation with Flanker concurrent task, measured with Flanker RT.

By grouping experimental subjects in clusters of typical versus non-typical digital EEG phenotypes, a differential response can be found in subjects with a non-typical electrophysiological profile. EEG profiles (summarized in Fig. 14) are characterized only by homogeneous resting-state EEG spectral features before each intervention. The clustering does not take into account the behavioral response to tasks. This confirms our hypothesis that the electrophysiological profile can be used as a proxy of different biophysical characteristics of the brain in experimental subjects. These characteristics are almost identical at 4 different measurement time points each taken a week apart as shown in the intra-subject variability analysis. Therefore we can state that the electrophysiological profiles are subject-specific and do not correspond to a difference in brain state. This is an important question given that brain state is known to influence as well the response to Non-Invasive Brain Stimulation^51^. We propose the use of the stratified electrophysiological responses as shown in this article as digital EEG phenotypes for the characterization of tDCS response.

It can be observed that Group B (i.e. receiving the stimulation over the rIFG brain area) is the only one with responders (both positive and negative), and Group A does not have any significant correlations supported with significant Wilcoxon-test results. This suggests an advantage in terms of post-tDCS task performance in stimulating the rIFG over the more traditional approach of lDLPFC stimulation, even when participant stratification is applied. In almost all groups of participants, the behavioral task showing changes is the Flanker task, when the applied stimulation montage targets enhancing the activity of the rIFG. This result is consistent with the current understanding of the role of the rIFG, associated with response inhibition and behavioral control^52,53^.

Future work could be done in order to establish a relationship between the electrophysiological profiles and functional outcomes. A hypothesis would be based on the transfer of stimulation effects from the stimulated working memory network components involved in the Flanker task to the working memory network components involved in the N-Back task^36,54–56^. There might be a response dependency due to the brain’s stage of development since, for example, Clusters 3 and 4 present different neurophysiological characteristics and show opposite results^36,57^.

In summary, we can state that the effect of brain stimulation (and its parameters including location and presence/absence of concurrent task during the protocol), measured by different behavioral measures, depends on the individual digital EEG phenotype of the subjects undergoing the stimulation intervention. Such a digital phenotype can be derived by applying the unsupervised clustering approach that we have developed. The robustness of our overall methodology was validated in 3 independent tests. First, the feature space of all participants was visualized showing that the features of all trials of pre-treatment EEG for each subject lie close to each other. Therefore, their baseline neurophysiological activity was similar over the different trials, which were conducted one week apart. Second, the k-hold-out methodology applied to the clustering pipeline demonstrates the robustness of the clustering algorithms in our specific dataset. This validation let us ensure to a certain extent the generalization capability of the clustering for generating similar digital EEG phenotypes on unseen data. Finally, the M-hold-out validatioin with $M=1000$ applied to the correlational and Wilcoxon test analysis shows a decreasing trend with decreasing sample sizes. Moreover it shows also that the correlational analysis and the behavioral response differences are robust enough to resist a reduction of 20% in the data set sample size.

Some limitations arise from the fact that the dataset is relatively small compared to the large amount of features analysed, resulting in a very high dimensional space for the employed clustering validity measures. This hinders to a certain extent the full automation of the methodology. In order to reduce the feature space dimensionality, Principal Feature Analysis^58^, permutation analysis^20^ and other feature selection algorithms^59,60^ could be explored to reduce the number of features. However the application of such approaches would decrease the explainability of the resulting methodology, i.e. digital phenotypes could not be given in the form of a complete electrophysiological characterization.

## Conclusions

The advanced clustering methodology we developed shows promising results in the context of broad and heterogeneous EEG signals^61^. Moreover the use of unsupervised learning approaches allows to obtain an actual data-driven stratification, since the learning procedure is freely run without the constraints imposed by the ground truth. This is an important factor given the large misdiagnosis rates in the target diseases^62,63^, especially important in ADHD^63^. In case of having used a supervised approach, a false ground truth might have driven the class structure wrongly and hinder the accuracy of the stratification.

The stimulation target areas studied were the lDLPFC and rIFG, paired or not with concurrent tasks N-Back and Flanker Tasks respectively. Effectiveness of the treatment was assessed by behavioral reaction times and accuracy, when performing experimental tasks after the tDCS treatment. The digital phenotypes obtained from pre-treatment EEG, as computed by the clustering procedure described herein, can then be used to predict the response of individuals to particular stimulation protocols. We have successfully developed a procedure for labelling clusters in terms of their behavioral response in the different tasks. Moreover, a rank-sum Wilcoxon test is successfully applied for validation and consistency of results.

Interestingly, positive responder groups correspond to participants with a non-typical brain profile, presenting either a slowing of the EEG or a spread of increased alpha rhythm over frontal areas. This finding is extremely interesting from a neurophysiological point of view. However, we focus in this communication on the methodological novelty of our work. A larger cohort of participants would be needed for confirming the clinical utility of the proposed approach, which we aim to achieve in future communications.

## Supplementary Information

Supplementary Information.

## Data Availability

Due to ethical restriction, the data from this study will not be able to be accessible from public domain. The data are available upon request. Vera Moliadze (STIPED consortium), Institute of Medical Psychology and Medical Sociology, University Medical Center Schleswig-Holstein, Kiel University, Kiel, Germany (moliadze@med-psych.uni-kiel.de).
