# Supplementary material for: Stratification of responses to tDCS intervention in a healthy pediatric population based on resting-state EEG profiles
Source: Sci Rep. 2023 May 25;13:8438. doi: 10.1038/s41598-023-34724-5 (PMC10213038; doi:10.1038/s41598-023-34724-5)
Supplement: Supplementary file 1 — Supplementary Information. [file 41598_2023_34724_MOESM1_ESM.pdf]

Stratification of responses to tDCS intervention in a healthy paediatric population based on resting-state EEG profiles

Paulina Clara Dagnino[1,+], Claire Braboszcz[1], Eleni Kroupi[1], Maike Splittgerber[2], Hannah Brauer[3], Astrid Dempfle[4], Carolin Breitling-Ziegler[5], Alexander Prehn-Kristensen[3], Kerstin Krauel[5], Michael Siniatchkin[6], Vera Moliadze[2], and Aureli Soria-Frisch[1,*,+]

[1]Starlab Barcelona SL, Neuroscience BU, Av Tibidabo 47 bis, Barcelona, Spain
[2]Institute of Medical Psychology and Medical Sociology, University Medical Center Schleswig-Holstein, Kiel University, Kiel, Germany
[3]Department of Child and Adolescent Psychiatry, Center for Integrative Psychiatry Kiel, University Medical Center Schleswig-Holstein, Kiel, Germany
[4]Institute of Medical Informatics and Statistics, University Hospital Schleswig Holstein, Kiel University, Kiel, Germany
[5]Department of Child and Adolescent Psychiatry and Psychotherapy, University of Magdeburg, Magdeburg, Germany
[6]Clinic for Child and Adolescent Psychiatry and Psychotherapy, Protestant Hospital Bethel, University of Bielefeld, Campus Bielefeld Bethel, Bielefeld, Germany
[*]Correspondence: aureli.soria-frisch@starlab.es
[+]these authors contributed equally to this work

Supplementary Information

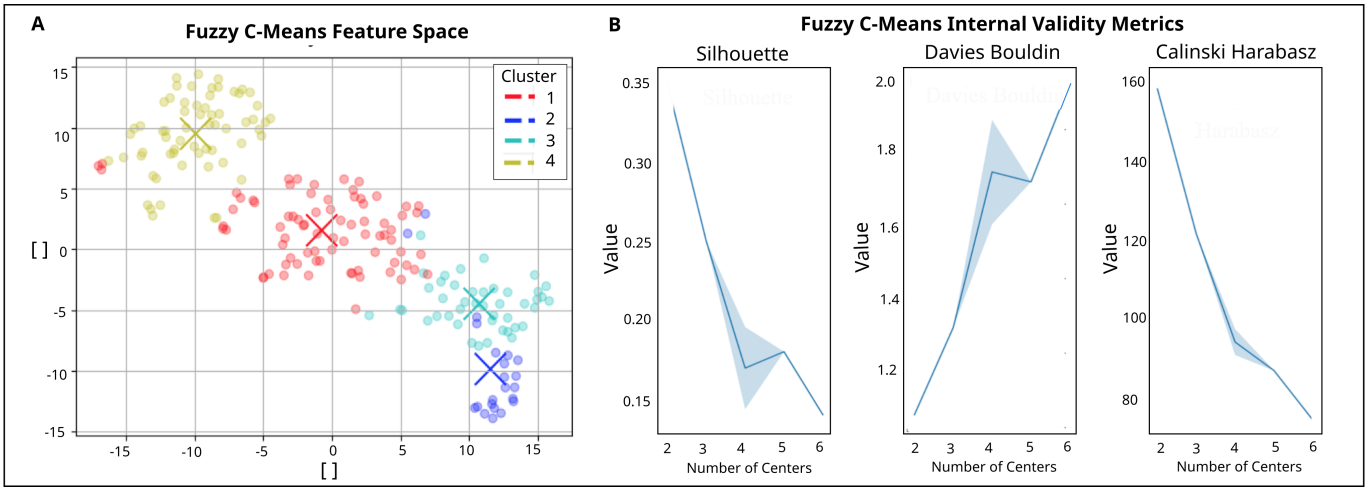

Supplementary Figure 1. Clustering Results for FCM Analysis. The left plot (A) shows the feature Space, colourcode represents each cluster. From all data-points, In FCM Clustering, 37.4% lie in Cluster 1, 10.7% in Cluster 2, 21.4% in Cluster 3 and 30.5% in Cluster 4. The right plots (B) show the evolution of the internal validity metrics Silhouette and Davies Bouldin when changing the number of centres. An elbow at *k*=5 can be seen.

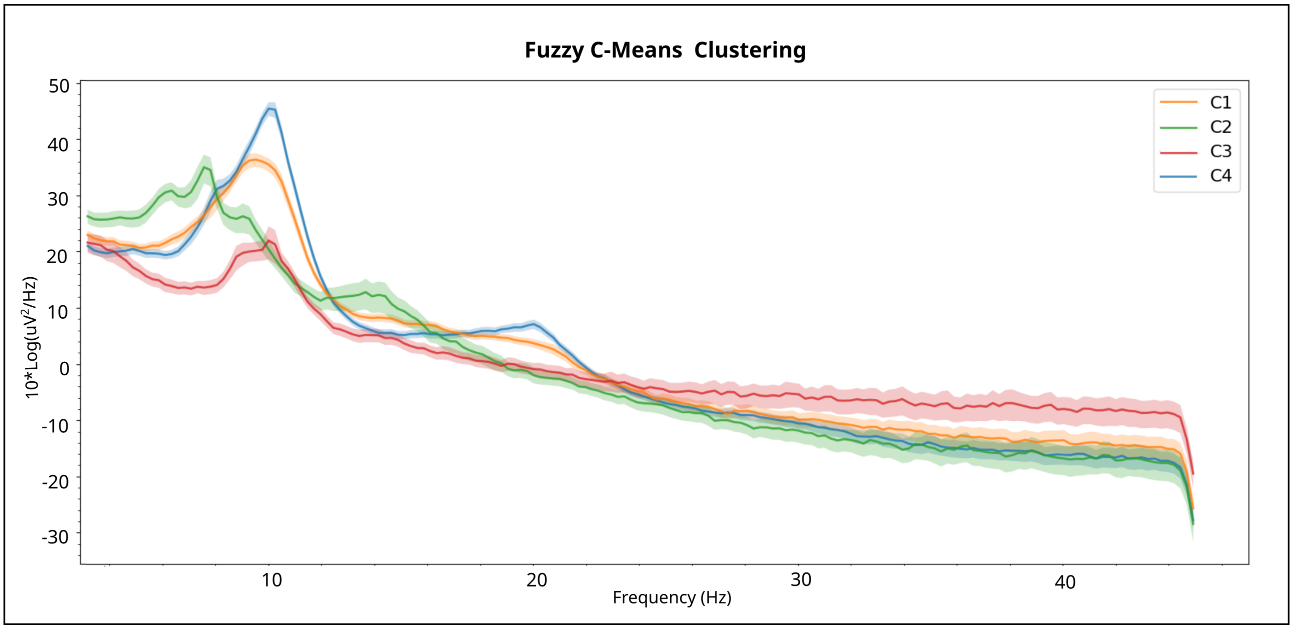

Supplementary Figure 2. Power Spectrum Plot of each Cluster for FCM Algorithm Results

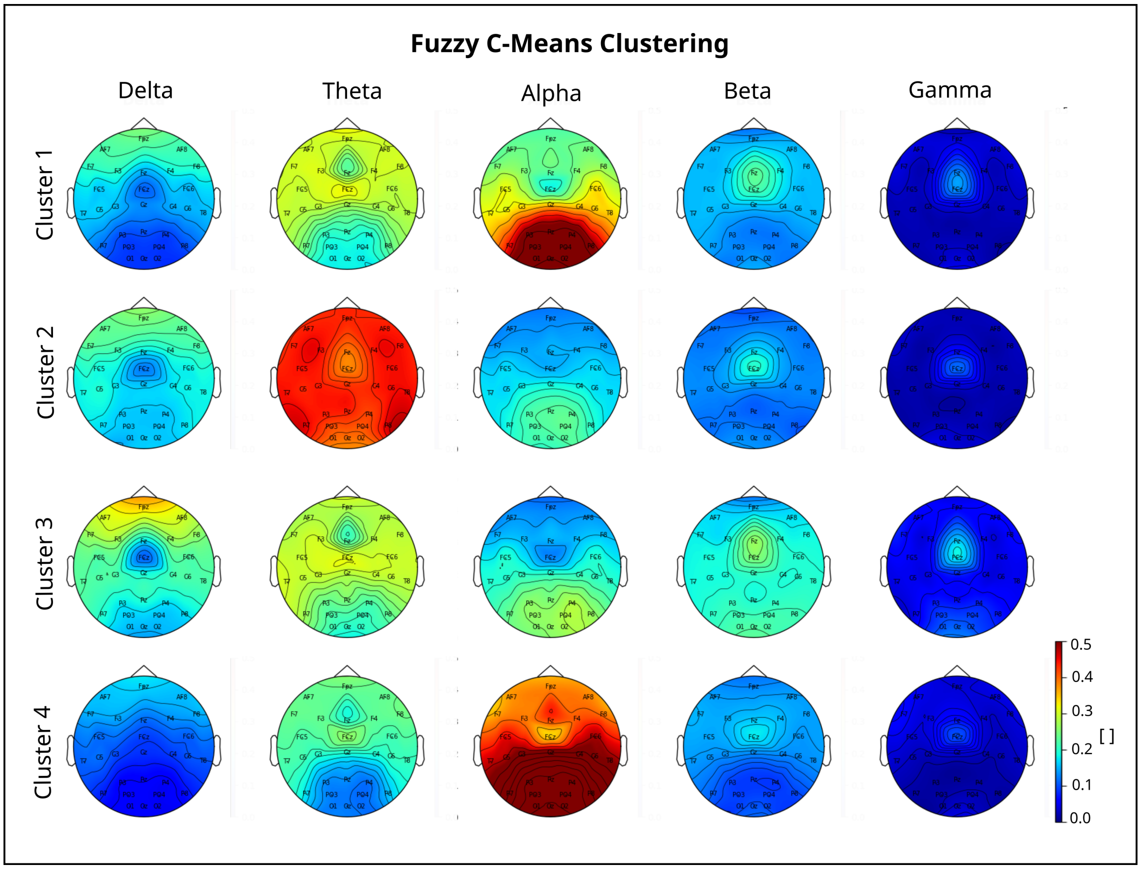

Supplementary Figure 3. **Power Spectrum Plot of each Cluster for FCM Algorithm Results**

Cluster	Group A, Concurrent	Group A, Non-concurrent	Group B, Concurrent	Group B, Non-concurrent
1				
2				N-Back Accuracy (+, 0.0287)
3				Flanker RT (-, 0.0245)
4				Flanker RT (+, 0.0287)

Supplementary Table 1. **FCM Significant Responders.** These results correspond to significant correlations and significant wilcoxon tests. In parenthesis are: (1) direction of correlation (positive or negative), (2) Wilcoxon p-values. There are significant correlations only in Group B, non-concurrent, therefore for Wilcoxon Test 5 corrections are done. Stimulation protocol Group B (rIFG), offline mode (non-concurrent task) presents positive response in Clusters 2 and 4 measured with N-Back Accuracy and Flanker RT respectively, and negative response in Cluster 3 assessed with Flanker RT.

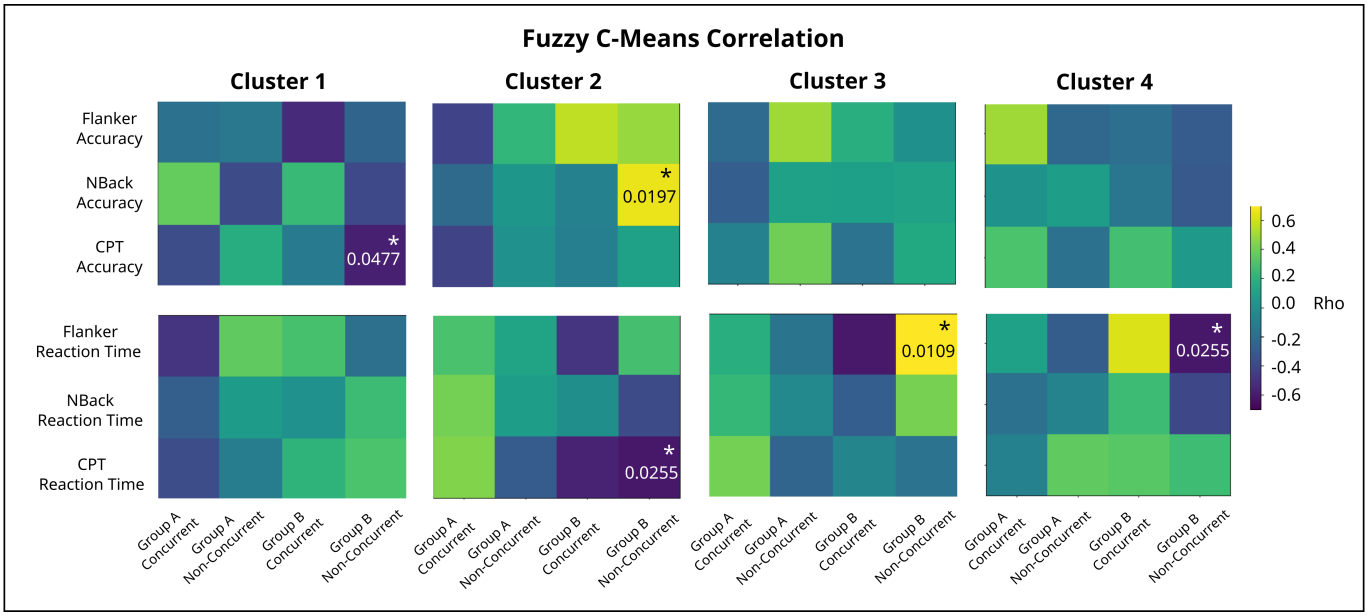

Supplementary Figure 4. Correlation Results for FCM. Each matrix row corresponds to a specific behavioural task endpoint, and each matrix column corresponds to a particular treatment protocol. The colourbar represents the sign of the correlation (positive or negative) and p-values are written in the significant cells. For Group B, 24 corrections are done; for Group A, 20 corrections are applied.

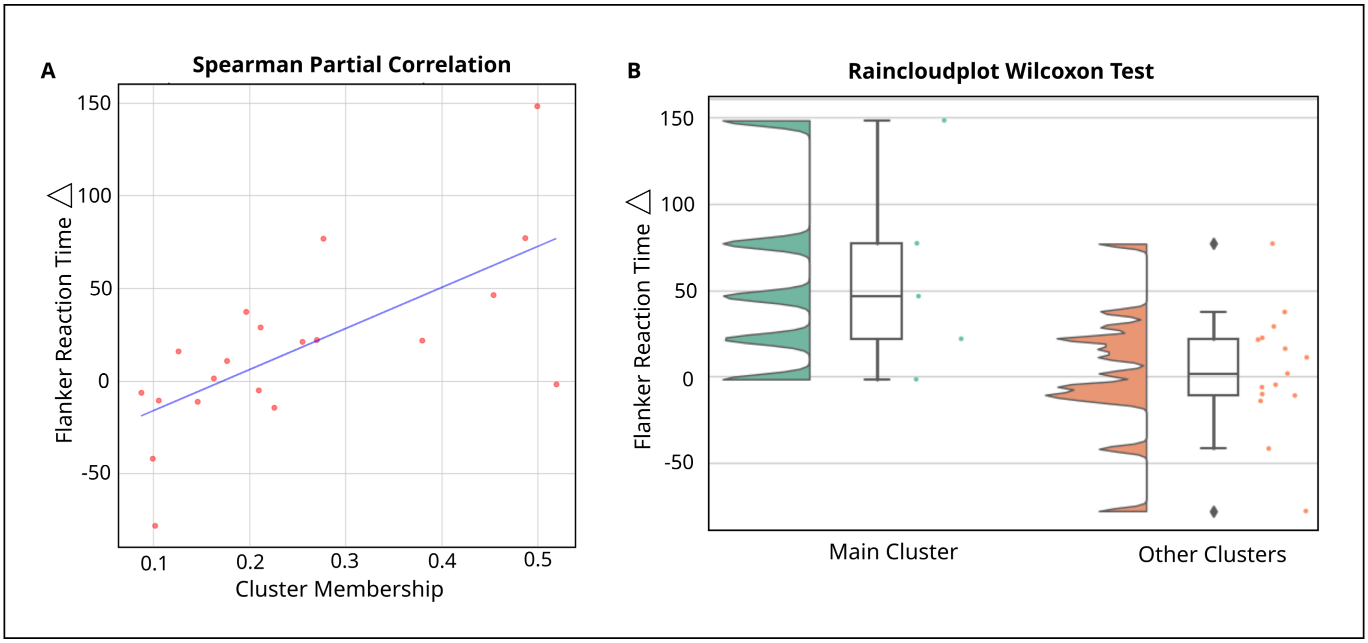

Supplementary Figure 5. Example of significant negative responder group for Spectral Clustering (Cluster 3), with rIFG stimulation with no concurrent task, measured with Flanker RT. (A) Correlation between cluster membership of pre treatment EEG spectral features of all participants and Flanker Reaction Time at the end of the trials. Best-fit line showing least square polynomial fit. (B) Wilcoxon Test between Flanker RT of Cluster 3 Spectral, and the rest of the Clusters. The raw data is visualized as points as well as the probability density, and key summary statistics in the boxplot (median, mean, and confidence intervals).

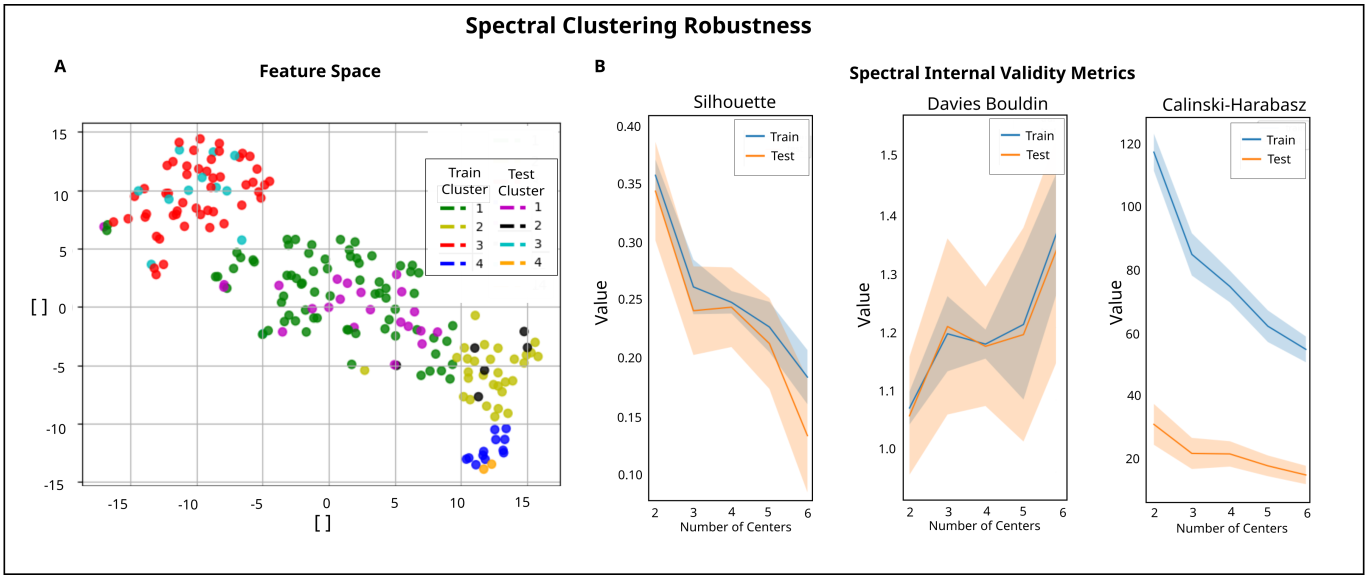

Supplementary Figure 6. Spectral Clustering Robustness via k-hold-out methodology. The left plot (A) shows the Feature Space after one iteration, colourcode represents each cluster for each set - train and test. Visual overlap is observed between the clusters assigned to each set. The right plots (B) show the evolution of the internal validity metrics Silhouette and Davies Bouldin when changing the number of centres after 50 iterations. The train and test sets show the same trend.

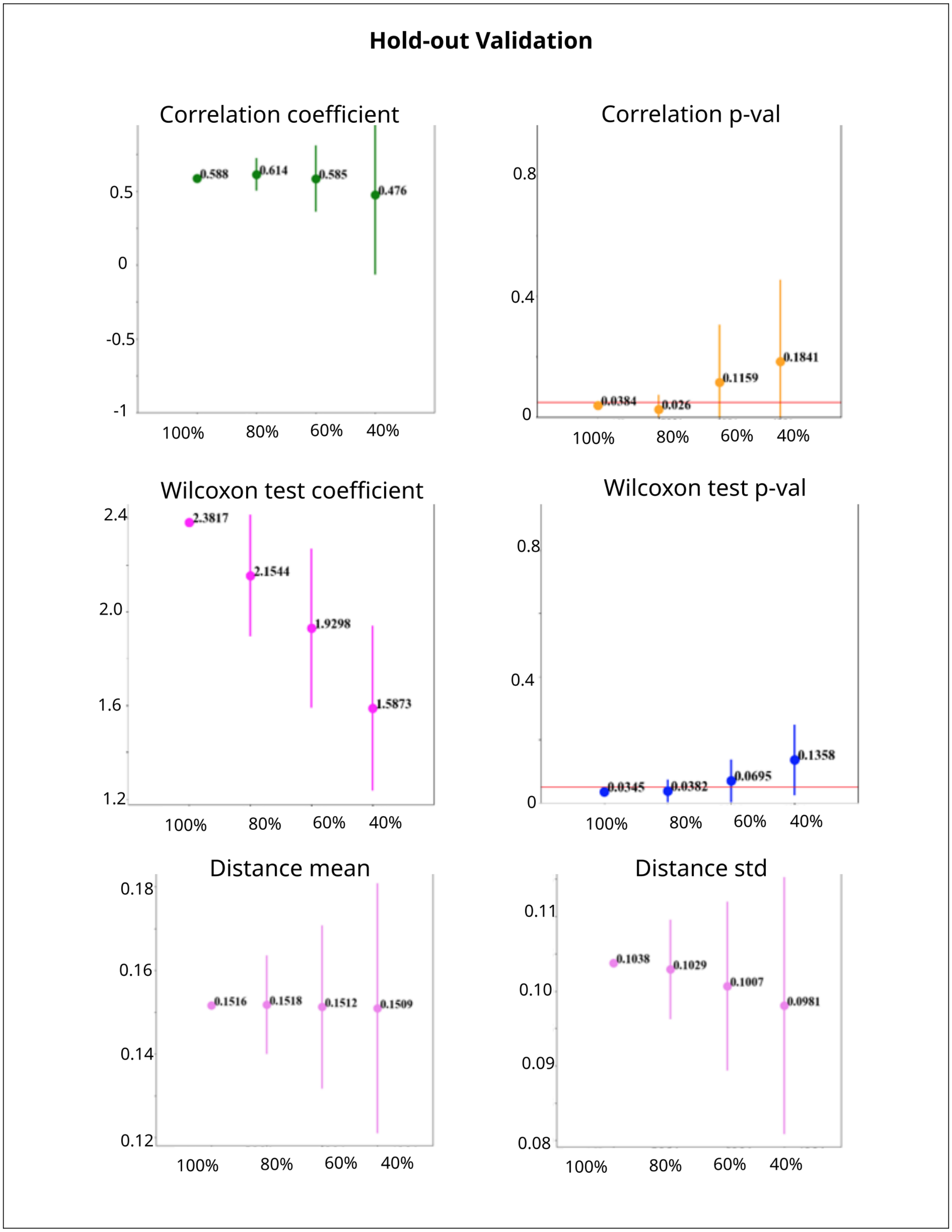

Supplementary Figure 7. Correlational analysis robustness for Cluster 2 of Spectral Clustering, with rIFG stimulation with no concurrent task, measured with N-Back Accuracy as a behavioral outcome measure. We have conducted a repeated hold-out validation, i.e. we have repeated the overall clustering, correlation and significance test with different percentages of the total number participants and repeated the procedure 1000 times in order to better estimate the average p-value and its variance. The plots show the estimated correlation and the p-value of the Wilcoxon test, as well as the distance to the original fit line, with respect to percentage of training. As we can observe both the correlation and the p-value are quite robust in spite of the reduced sample size.

Subject ID	Group	Sex	Age
1f	A	1	15
2m	A	2	17
3f	A	1	15
4m	A	2	14
5m	A	2	16
5f	A	1	17
7m	A	2	13
7f	A	1	14
10f	A	1	17
11f	A	1	10
12m	A	2	16
13f	A	1	13
14m	A	2	15
15m	A	2	11
15f	A	1	14
17f	A	1	14
17m	A	2	11
19f	A	1	17
22m	A	2	13
25f	A	1	16
26m	A	2	10
27m	A	2	12
28f	A	1	16
29f	A	1	16
31f	A	1	15
33f	A	1	16
35f	A	1	13
3m	B	2	17
4f	B	1	14
6f	B	1	14
6m	B	2	15
8m	B	2	17
8f	B	1	17
9f	B	1	13
10m	B	2	13
11m	B	2	17
12f	B	1	12
13m	B	2	15
14f	B	1	15
16m	B	2	11
16f	B	1	17
18f	B	1	17
18m	B	2	10
19m	B	2	14
20f	B	1	13
21m	B	2	11
22f	B	1	14
24f	B	1	14
24m	B	2	11
25m	B	2	17
26f	B	1	14
27f	B	1	14
28m	B	2	12
30f	B	1	13
32f	B	1	14
34f	B	1	17

Supplementary Table 2. Clustering participant information. The table shows ID, Group (A and B), sex (1: female, 2: male) and age.

Subject ID	IQ	Left EHI	Right EHI
1f	108	1	19
2m	101	1	12
3f	98	3	12
4m	108	2	9
5m	94	2	10
5f	97	1	13
7m	99	19	1
7f	107	10	0
10f	119	1	19
11f	110	20	0
12m	103	2	16
13f	100	0	10
14m	99	0	10
15m	109	3	10
15f	103	0	10
17f	102	0	10
19f	127	0	10
25f	116	12	5
26m	119	0	20
28f	98	1	10
29f	99	4	16
31f	111	5	5
33f	107	0	16
35f	120	12	5
3m	127	3	13
4f	84	0	10
6f	104	0	10
8m	115	3	10
8f	94	2	10
9f	102	0	14
10m	97	0	11
12f	115	1	10
14f	101	10	1
16m	129	4	10
16f	101	0	10
18f	115	4	12
20f	109	10	0
22f	97	1	10
25m	115	0	20
26f	103	1	9
28m	110	2	12
30f	91	3	12
32f	88	2	10
34f	80	0	12

Supplementary Table 3. Correlation Participant information. The table shows ID, IQ and handedness measured with the Edinburgh Handedness Inventory (EHI).
